# Characterisation of a Hydroxycinnamic Acid Esterase From the *Bifidobacterium longum* subsp. *longum* Taxon

**DOI:** 10.3389/fmicb.2018.02690

**Published:** 2018-11-09

**Authors:** Sandra M. Kelly, John O’Callaghan, Mike Kinsella, Douwe van Sinderen

**Affiliations:** ^1^School of Microbiology, University College Cork, Cork, Ireland; ^2^APC Microbiome Ireland, University College Cork, Cork, Ireland; ^3^Pharmaceutical and Molecular Biotechnology Research Centre, Department of Science, Waterford Institute of Technology, Waterford, Ireland

**Keywords:** esterase, hydroxycinnamic acids, plant phenolics, ferulic acid, *p*–coumaric acid, caffeic acid, bifidobacteria

## Abstract

*Bifidobacterium longum* subsp. *longum*, a common member of the human gut microbiota with perceived positive health effects, is capable of metabolising certain complex, plant-derived carbohydrates which are commonly found in the (adult) human diet. These plant glycans may be employed to favourably modulate the microbial communities in the intestine. Hydroxycinnamic acids (HCAs) are plant phenolic compounds, which are attached to glycans, and which are associated with anti-oxidant and other beneficial properties. However, very little information is available regarding metabolism of HCA-containing glycans by bifidobacteria. In the current study, a gene encoding a hydroxycinnamic acid esterase was found to be conserved across the *B. longum* subsp. *longum* taxon and was present in a conserved locus associated with plant carbohydrate utilisation. The esterase was shown to be active against various HCA-containing substrates and was biochemically characterised in terms of substrate preference, and pH and temperature optima of the enzyme. This novel hydroxycinnamic acid esterase is presumed to be responsible for the release of HCAs from plant-based dietary sources, a process that may have benefits for the gut environment and thus host health.

## Introduction

Bifidobacteria are Gram-positive gut commensals of various mammals, insects and birds, where their presence is associated with a number of beneficial effects (Ventura et al., 2014). Such beneficial effects include pathogen inhibition (Fukuda et al., 2011; Vazquez-Gutierrez et al., 2016), immune modulation (Fanning et al., 2012; Turroni et al., 2013), reduction in the symptoms of irritable bowel syndrome (Whorwell et al., 2006) and cholesterol reduction (Zanotti et al., 2015). In humans, bifidobacteria are particularly abundant and prevalent in the infant gut, though their relative abundance reduces upon weaning and upon ageing of their host (Turroni et al., 2012). Members of the *Bifidobacterium* genus commonly metabolise a range of dietary and host-derived carbohydrates, with the precise substrate nature of this versatile carbohydrate metabolism being strain/species specific (Pokusaeva et al., 2011). The ability to metabolise diet- and host-derived carbohydrates is believed to enable efficient bifidobacterial gut colonisation and persistence. For example, infant-associated bifidobacterial species/strains can typically metabolise human milk oligosaccharides (HMO) present in breast milk, while species/strains found in adults tend to metabolise various dietary plant polysaccharides (Schell et al., 2002; LoCascio et al., 2007; James et al., 2016; Maldonado-Gomez et al., 2016).

Members of the *Bifidobacterium longum* subsp. *longum* taxon have the capacity to metabolise various plant cell wall polysaccharides, such as arabinoxylan, and pectic components, such as arabinan (Schell et al., 2002; Al-Tamimi et al., 2006; Onumpai et al., 2011; Van Den Abbeele et al., 2013; Riviere et al., 2014; Moon et al., 2015; O’Callaghan et al., 2015; Arboleya et al., 2018). Therefore, these polymeric glycans are considered suitable substrates to stimulate growth of *B. longum* subsp. *longum* in the adult gut. Plant phenolic compounds, such as ferulic acid and *p*–coumaric acid, also sometimes referred to as hydroxycinnamic acids (HCAs), may be present as substitutes to the L-arabinose moieties of such plant polymers by means of ester linkages (Fry, 1982; Smith and Hartley, 1983). Although various studies have described aspects of arabinoxylan and arabinan metabolism, little is known about HCA metabolism by bifidobacteria.

Hydroxycinnamic acids are commonly found in various foods, being present in cereals, fruit, vegetables and coffee, among others (Hanhineva et al., 2010; Mills et al., 2013). HCAs have been associated with a variety of beneficial effects, including diabetes resistance in rats (Lai et al., 2009), intestinal pathogen inhibition (Lee et al., 2006), inhibition of platelet aggregation (Fuentes et al., 2013), anti-oxidant and anti-carcinogenic activities (Huang et al., 1988). Several studies have shown that certain fungi possess HCA esterases with broad substrate specificity, while more recently it has been demonstrated that bacterial species, including lactobacilli and bifidobacteria, produce esterases that cleave artificial HCA-containing substrates and are (presumed to be) capable of removing HCAs from plant substrates (Castanares et al., 1992; Wang et al., 2004; Hassan and Hugouvieux-Cotte-Pattat, 2011; Esteban-Torres et al., 2013; Raimondi et al., 2015; Xu et al., 2017). Therefore, gut commensals that produce HCA-active esterases are believed to play a role in releasing HCAs from plant carbohydrates. It may be that HCAs must be first removed from the plant carbohydrate to give access to other enzymes involved in plant carbohydrate degradation. The purpose of HCA release may also provide an energy advantage to bacteria as it has been shown HCAs can be used as external electron acceptors (Filannino et al., 2014, 2016). Furthermore, HCAs can inhibit growth of spoilage bacteria in high concentrations and HCA metabolism is thought to detoxify inhibitory HCAs (Sánchez-Maldonado et al., 2011).

Esterases and lipases are two important groups within the hydrolase class of enzymes. Both esterases and lipases cleave ester bonds, possess an α/β hydrolase fold and generally exhibit a consensus sequence of (Gly-X–Ser–X–Gly) around the catalytic triad residues Ser–His–Asp (Kroon et al., 2000; Bornscheuer, 2002). Esterases, in contrast to lipases, generally follow Michaelis-Menten kinetics and hydrolyse substrates that are less than six carbons in length (Arpigny and Jaeger, 1999; Bornscheuer, 2002). Esterases can also be categorised into four groups based on substrate preference (Crepin et al., 2004).

In the current study we identified a hydroxycinnamoyl acid esterase-encoding gene, designated *caeA*, in the genome of members of the *B. longum* subsp. *longum* taxon, positioned within a conserved locus predicted to be associated with arabinoxylan and arabinan metabolism. Heterologous expression, purification and subsequent characterisation of the CaeA protein demonstrated that it indeed represents a genuine esterase, as opposed to a lipase, and can cleave several HCA-containing substrates. The biochemical properties of the CaeA esterase were investigated and the optimal enzyme pH and temperature ascertained. Therefore, this hydroxycinnamic esterase is another *B. longum* subsp. *longum* enzyme that may contribute to this taxon’s ability to metabolise plant-derived polysaccharides.

## Materials and Methods

### Bacterial Strains, Plasmids, Growth Conditions and Chemicals

Bacterial strains and plasmids used in this study are summarised in Table 1. Bifidobacteria were routinely cultured on Reinforced Clostridium Agar (RCA) or in modified deMan, Rogosa, Sharpe medium (mMRS) supplemented with 1 % (w/v) lactose (Sigma-Aldrich, Steinheim, Germany) and 0.05% (w/v) cysteine–HCL (Sigma-Aldrich) (Man et al., 1960). All bifidobacteria were cultivated under anaerobic conditions in a modular atmosphere-controlled system (Davidson and Hardy, Belfast, United Kingdom). *Lactococcus lactis* strains were grown in M17 broth (Oxoid, Basingstoke, Hampshire, United Kingdom) supplemented with 0.5% (w/v) glucose at 30°C. Where required media was supplemented with 5 mg ml-1 chloramphenicol. For RCA ethyl ferulate plate assays, RCA medium was supplemented with 0.1% (v/v) ethyl ferulate dissolved in 96% ethanol. Methyl ferulate, ethyl ferulate, methyl *p*–coumaric acid, methyl sinapinate, methyl caffeic acid (caffeate), and feruloyl glucose were all dissolved in 96% ethanol (Carbon Chemicals, Ringaskiddy, Ireland) and sourced from Carbosynth, Berkshire, United Kingdom. Para-nitrophenol (*p*-Np) acetate, *p*-Np butyrate, *p*-Np octanoate and *p*-Np dodecanoate were purchased from Sigma-Aldrich. All ions were purchased from Sigma-Aldrich.

**Table 1:** Bacterial strains and plasmids used in this study.

Bacterial strain/plasmid	Features	Reference
***Lactococcus*** ***lactis***		
NZ9000	MG1363 *pepN::nisRK;* nisin inducible overexpression host	de Ruyter et al., 1996
NZ9000-pNZ8150	NZ9000 containing plasmid pNZ8150	Mierau and Kleerebezem, 2005
NZ9000-pNZcaeA-His	NZ9000 containing pNZ8150 expressing *caeA* with an in frame His tag	This study
***Bifidobacterium*** ***longum*** ***subsp***. ***longum***		
NCIMB 8809	Nursling stool isolate	NCIMB, Aberdeen, Scotland
***Bifidobacterium breve***		
UCC2003	Nursling stool isolate	Maze et al., 2007
UCC2003-pNZ44caeA	UCC2003 containing pNZ44caeA	This study
**Plasmids**		
pNZ8150	Cm^R^, translational fusion vector induced by nisin.	Mierau and Kleerebezem, 2005
pNZcaeA-His	*caeA* with a His tag cloned downstream of the nisin inducible promoter on pNZ8150.	This study
pNZ44	Cm^R^, derivative of pNZ8048 with a constitutive promoter.	McGrath et al., 2001
pNZ44caeA	Cm^R^, pNZ44 derivative containing *caeA*	This study


### Nucleotide Sequence Analysis

Bacterial genomes were assessed using the Artemis genome browser (Rutherford et al., 2000) employing the annotated genome of *B. longum* subsp. *longum* NCIMB 8809 genome (O’Callaghan et al., 2015). Nucleotide analysis was completed using the programmes SeqMan and SeqBuilder of DNASTAR software (DNASTAR, Madison, WI, United States). Annotation of protein function and HMM-HMM homology detection, both under standard settings, were determined using BlastP and HHPred, respectively (Altschul et al., 1990, 1997;Söding et al., 2005; Alva et al., 2016). Protein alignments were generated using Clustal omega (Sievers et al., 2011).

### DNA Manipulations

All DNA manipulations were carried out as previously described (Sambrook and Russell, 2006). Chromosomal DNA was isolated from *B. longum* subsp. *longum* NCIMB 8809 using the GenElute Bacterial Genomic DNA Kit (Sigma-Aldrich). Primers for genomic amplifications were synthesised by Eurofins (Ebersburg, Germany). Genomic PCR reactions were performed with Q5 High-Fidelity 2X Master Mix (New England Biolabs, Herefordshire, United Kingdom) or Taq PCR master mix (Qiagen GmbH, Hilden, Germany). PCR products were cleaned using the Roche High Pure PCR Kit (Roche Diagnostics, Basel, Switzerland). Plasmid DNA was isolated using the High Pure Plasmid Prep Kit (Roche Diagnostics) with an added initial step of incubating resuspended cells with 30 mg ml^-1^ lysozyme (Sigma-Aldrich) at 37°C for 30 min. Restriction enzymes (Roche Diagnostics) and T4 (Promega) were used as per manufacturer’s instructions. Colony PCR was performed using Extensor Hi-Fidelity PCR Master mix (Thermo Fisher Scientific, Waltham, MA, United States). DNA electroporation procedures for *B. breve* and *L. lactis* were as previously described (Wells et al., 1993; Maze et al., 2007). The integrity of all constructs was confirmed by DNA sequencing (performed by Eurofins, Ebersburg, Germany).

### Plasmid Construction for Heterologous Expression of *caeA* in *B. breve* UCC2003

To construct the pNZ44caeA overexpression plasmid, the gene encoding the cinnamoyl esterase (B8809_1755), designated here as *caeA*, was amplified from the genomic DNA of *B. longum* subsp. *longum* NCIMB 8809 as a template using the polymerase Taq PCR master mix and primers CaeAF and CaeAR. Primer sequences used in this study are listed in Table 2. The generated PCR amplicon was restricted using NcoI and XbaI, and ligated to pNZ44 that had been similarly restricted with NcoI and XbaI. The resulting plasmid construct was electroporated into *L. lactis* NZ9000 and transformants were selected for by Cm^R^ resistance. Transformants containing the desired recombinant plasmid were confirmed by colony PCR using Extensor PCR Master Mix. Plasmid DNA was extracted from such transformants and clone integrity was confirmed by sequencing, resulting in plasmid pNZ44caeA, which was then electroporated into *B. breve* UCC2003 using chloramphenicol selection.

**Table 2:** Oligonucleotide sequences used in this study.

Function	Primer	Sequence
Cloning of *caeA* into pNZ44	CaeAF	gctcga **ccatgg** atcagcgt tcatcattcg^∗^
Cloning of *caeA* into pNZ44	CaeAR	ctctgc **tctaga** gaatgtccgc gcagccgtac
Cloning of *caeA* with His tag into pNZ8150	CaeAHisF	cctgca **gatatc** atgcatcaccat caccatcacca tcaccatcacgacat caaaccgtgggaatac
Cloning of *caeA* with His tag into pNZ8150	CaeAHisR	ctctgc **tctaga** gaatgtccgc gcagcc gtac


*^∗^Restriction enzyme sites are highlighted in bold.*

### Ethyl Ferulate Plate Assay

The ethyl ferulate plate assay was carried out as described previously with modifications (Donaghy et al., 1998). Bacterial cultures were grown in mMRS supplemented with 1 % (w/v) lactose overnight and were spot plated (10 μl) on to RCA with 0.1% (v/v) ethyl ferulate. Plates were then incubated anaerobically for 72 h at 37°C. A zone of clearing on the RCA ethyl ferulate plate around the colonies was taken as an indication of esterase activity.

### Expression and Purification of CaeA in *L. lactis* NZ9000

To construct the pNZcaeA-His plasmid to achieve overexpression and purification of His-tagged CaeA, primers CaeAHisF, which contained a sequence to add an in-frame N-terminal His-10 tag to the encoded CaeA protein, and CaeAHisR were used to amplify *caeA* from the genomic DNA template of *B. longum* subsp. *longum* NCIMB 8809 using Taq PCR master mix. The generated amplicon was digested with EcoRV and XbaI, and ligated to pNZ8150 digested with ScaI and XbaI. The ligation mixture was introduced into *L. lactis* NZ9000 by electroporation with Cm^R^ selection and positive clones were confirmed by colony PCR using Extensor PCR Master Mix and recombinant plasmid integrity was confirmed by DNA sequencing. For overexpression, 400 ml of M17 broth supplemented with 0.5% glucose was inoculated (2% v/v) with *L. lactis* NZ9000-pNZcaeA-His and incubated at 30°C until an OD_600nm_ of 0.5 was reached. Protein production was induced with purified nisin (5 ng ml^-1^) for 2 h. Cells were then harvested by centrifugation and the His-tagged CaeA protein was purified using the PrepEase His-tag protein purification kit (USB, Germany). Protein eluate fractions were analysed by SDS-polyacrylamide gel electrophoresis on a 12.5% polyacrylamide gel (Laemmli, 1970) with the Color Prestained Protein Standard, Broad Range (11–245 kDa) ladder (New England BioLabs, United States). Polyacrylamide gels were then fixed and stained using a Coomassie Brilliant Blue to indicate which fractions contained the purified protein. Protein aliquots were dialysed overnight in 50 mM NaH_2_PO_4_/K_2_HPO_4_ buffer pH 7 using dialysis tubing (Medicell Membranes Ltd., London, United Kingdom) to remove imidazole remaining from the protein purification. The amount of protein in each aliquot was determined by the Bradford Assay (Sigma-Aldrich) after dialysis (Bradford, 1976).

### HPLC Reactions

For High Performance Liquid Chromatography (HPLC) reactions, potential substrates methyl ferulate, ethyl ferulate, methyl *p*–coumaric acid, methyl sinapinate and methyl caffeic acid were dissolved in 96% ethanol to generate 20 mM stock solutions. Reactions were carried out in 20 mM morpholinepropanesulfonic acid (MOPS) pH 7.5 with the substrates present at a 1 mM final concentration and 15 μg of CaeA protein per reaction in a final reaction volume of 1 ml. Potential substrates were also incubated in buffer without CaeA as a negative control. All reactions and negative controls were incubated at 37°C for 16 h and were terminated by the addition of 370 μl ethyl acetate (Fisher Scientific) followed by centrifugation at 12,000 × *g*. The upper phase was then removed to a new tube and a further 370 μl of ethyl acetate was added, followed by mixing and centrifugation at 12,000 × *g*. This second extraction was then used for analysis. The cinnamic acids and esters were detected, separated on an Agilent 1200 series LC instrument coupled with an MSD Trap XCT Ultra Ion Trap mass spectrometer. Mobile phase A consisted of water + 0.1% formic acid and mobile phase B consisted of Acetonitrile + 0.1% formic acid. A highly refined and optimised gradient method was developed to separate all of the cinnamic acids and esters, and this was achieved in a 47 min run. The chromatography column used for separation was an Agilent Eclipse XDB C-18 column (150 mm × 4.6 mm), and the column oven was maintained at 40°C. An injection volume of 5 μL was used for all injections with ethanol used as a needle wash and UV detection was completed in parallel to mass spectrometry as a detection system. UV wavelengths of 280 and 320 nm were selected for measurement purposes.

For mass spectrometry-based detection, positive alternating mode was used, acquiring data in both positive and negative mode, though in general the detected analytes were more suited to negative mode analysis. A scan range of 100 – 2200 m/z was used with a capillary voltage of -3500 V, Nebuliser pressure of 50 psi, Dry gas (Nitrogen) was utilised at 10 L/minute, a drying temperature of 350°C was used and an m/z value of 220 was employed as the set target mass. The skimmer was set to 40 V, while the capillary exit was at 107.5 V.

### Substrate Specificity Assay

Enzyme reactions were carried out at 37°C in 0.1 M NaH_2_PO_4_/K_2_HPO_4_ buffer containing 0.6% (v/v) Triton – X – 100 and 1.1 mg/ml of gum arabic (Sigma – Aldrich) at pH 7.5. 20 mM stock solutions of *p*-Np acetate, *p*-Np butyrate, *p*-Np octanoate, and *p*-Np dodecanoate were prepared in 1:4 (v/v) acetonitrile: isopropanol. All reactions had a final substrate concentration of 6 or 12 μg/ml CaeA protein in a final reaction volume of 1 ml. Esterase enzymatic activity was measured by the release of *p*-Np from the substrates at the pH-independent wavelength 348nm. Reactions were terminated after 30 s by the addition of 25 μl of concentrated HCl (36%) (Sigma-Aldrich). The rate of enzyme activity was calculated as μmol min^-1^mg^-1^ of *p-*Np released. The maximal enzyme activity observed was then defined as 100% and the relative activity for each reaction was calculated accordingly.

Hydrolysis of ethyl ferulate, methyl ferulate, methyl *p*-coumaric, methyl sinapinate or methyl caffeate was determined using *p*-Np as a proton sink as previously described with modifications (Janes et al., 1998). A 10 mM stock solution of each substrate dissolved in 96% ethanol was prepared. A 10 mM stock solution of *p*-Np (Sigma-Aldrich) was used to prepare 1 mM NaH_2_PO_4_/K_2_HPO_4_ buffer (pH 7) with *p*-Np at a final concentration of 0.44 mM. Assays were carried out in this buffer with 6 or 12 μg/ml CaeA and substrates at a final concentration of 1 mM in 200 μl at 37°C for 2 h. The rate of the enzyme activity was calculated as μmol min^-1^mg^-1^ of HCA released with standard curves for each corresponding HCA.

### Optimal Temperature, pH and Ion Assay

A 20 mM stock solution of *p*-Np butyrate substrate was prepared in 1:4 (v/v) acetonitrile: isopropanol and 0.3% (v/v) Triton – X – 100 (All from Sigma-Aldrich). Enzymatic assays were performed at 20, 25, 30, 37, 40, 50, 55°C for 30 s in 0.1 M NaH_2_PO_4_/K_2_HPO_4_ buffer at pH 7.5 with 6 μg/ml CaeA protein and a final concentration *p*-Np butyrate of 2 mM (100 μl) in a total reaction volume of 1 ml.

For optimum pH assays, a stock of 20 mM *p*-Np butyrate was prepared in 1:4 (v/v) acetonitrile: isopropanol. Impact of pH on enzyme activity was determined at 37°C in 0.2 M citric acid phosphate buffer (pH 3 – 5), 0.1 M NaH_2_PO_4_/K_2_HPO_4_ buffer (pH 5 – 8) and 50 mM Tris HCL (7 – 9). All buffers also contained 0.6% (v/v) Triton – X – 100 and 1.1 mg/ml gum arabic. The pH-variable assays were performed for 30 s with 6 or 12 μg/ml of protein with a final concentration of *p*-Np butyrate of 2 mM in a total reaction volume of 1 ml. For both assays, rate of enzyme activity was calculated as μmol min^-1^mg^-1^ of *p-*Np released. The maximal enzyme activity was then defined as 100% and relative activity for each reaction was calculated. Enzymatic activity was measured at the pH independent wavelength 348 nms.

The effect of metal ions on enzyme activity was also tested. Enzyme reactions were carried out at 37°C in 0.1 M NaH_2_PO_4_/K_2_HPO_4_ buffer pH 7.5 in a microtiter plate. A stock of 20 mM of each ion was prepared in water. A stock of 20 mM *p*-Np butyrate was prepared in 1:4 (v/v) acetonitrile:isopropanol. Assays were performed in a final volume of 200 μl for 10 min with a final concentration of 6 μg/ml of protein and 2 mM *p*–Np butyrate. Ions were at a final concentration of 1 mM. Enzymatic activity was measured in all assays by the release of *p*–Np at the pH-independent wavelength of 348 nm after 10 min. The rate of the enzyme activity was calculated as μmol min^-1^mg^-1^ of *p-*Np released. The maximal enzyme activity was then defined as 100% and relative activity for each reaction was then calculated.

### HPAEC-PAD Analysis

The feruloyl glucose substrate was dissolved in ethanol. Reactions were carried out in 0.1 M sodium phosphate pH 7.5 with the substrate at a 0.5 mg/ml final concentration and 15 μg of CaeA in a final reaction volume of 1 ml. A negative control including just feruloyl glucose and buffer (i.e., without enzyme) was also performed. Reactions and negative controls were incubated at 37°C for 16 h and terminated by heating the sample at 98°C for 2 min. Standard solutions of 1 mg/ml glucose prepared in water and 0.5 mg/ml feruloyl glucose in ethanol were used. Standards and reactions were freshly prepared immediately prior to analysis. Samples were stored at 4°C before their assessment by High-Performance Anion Exchange Chromatography – Pulsed Amperometric Detection (HPAEC-PAD) analysis, which was performed employing a Dionex ICS-3000 system (Sunnyvale, CA, United States) as follows. A 25 μl aliquot of each of the esterase reactions was separated on a CarboPac PA1 analytical exchange column (250 mm × 4 mm) with a CarboPAC PA1 guard column (50 mm × 4 mm) and a pulsed electrochemical detector (ED40) in the PAD mode. All columns and detectors were acquired from Dionex. Elution was carried out at a constant flow-rate of 1.0 ml min^-1^ at 30°C using the following eluents: eluent A, 200 mM NaOH; eluent B, 100 mM NaOH with 550 mM Na acetate and eluent C, MilliQ water. Analysis was performed using a linear gradient of sodium acetate with 100 mM NaOH from 0 min to 50 min, 0 mM; from 50 to 51 min, 100 mM; from 56 to 61 min.

## Results

### Identification of an Esterase-Encoding Gene Conserved Among Members of the *B. longum subsp. *longum** Taxon

**FIGURE 1:**
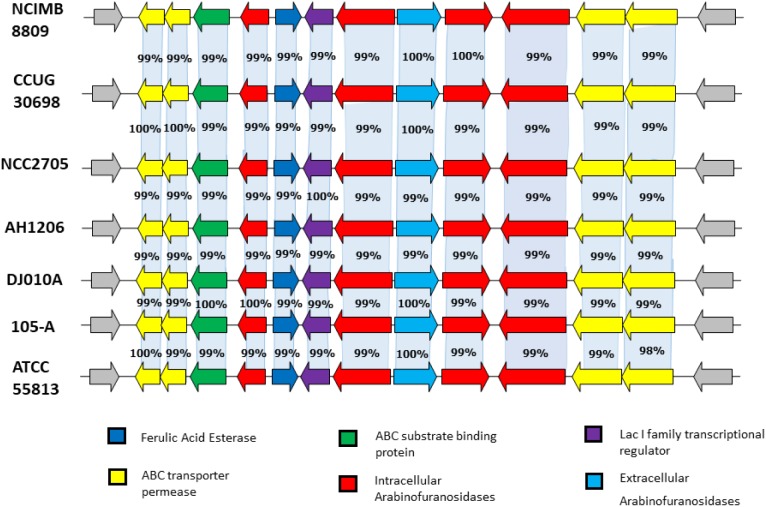
Comparison of the conserved plant oligosaccharide degradation locus amongst complete and available genomes of the *B. longum* subsp. *longum* taxon. *B. longum* subsp. *longum* strains are indicated in bold. The arrows represent open reading frames which are proportional to open reading frame length. The nucleotide identity of each of the open reading frames is calculated as a percentage of identity to the equivalent open reading frame in *B. longum* subsp. *longum* NCIMB 8809.

*Bifidobacterium longum* subsp. *longum* has the capacity to metabolise plant carbohydrates; for example, arabinofuranosidases have been annotated and/or studied from strains in this taxon (Schell et al., 2002; Margolles and De Los Reyes-Gavilán, 2003; Gueimonde et al., 2007). However, no esterases, enzymes involved with the removal of HCAs from plant phenolics, have curently been studied from this taxon. An *in silico* search for an esterase gene in the available genome sequences of members of the *B. longum* subsp. *longum* taxon using Blastn revealed a highly conserved locus (B8809_1751 – B8809_1762 in *B. longum* subsp. *longum* NCIMB8809), predicted to be involved in plant-derived oligosaccharide degradation within the *B. longum* subsp. *longum* taxon (Schell et al., 2002; Arboleya et al., 2018; Arzamasov et al., 2018) (Figure 1). The locus includes genes predicted to encode (i) five arabinofuranosidases (B8809_1754, B8809_1757 – BB8809_1760), enzymes that are known to release arabinose moieties from certain plant polysaccharides such as arabinoxylan and arabinan; (ii) four ABC transporter permeases and a solute binding protein, which are presumed to be involved in the transport of arabinose into the cell (BB8809_1751 – 1753, BB8809_1761-1762); (iii) an esterase (BB8809_1755), and (iv) a LacI-type regulatory protein (B8809_1756), which may be responsible for transcriptional control of the genes of this locus. The gene product of B8809_1754, or AbfII2 as previously designated, exhibits 51% similarity to a previously characterised arabinofuranosidase from *Streptomyces avermitilis* NBRC14893 (Ichinose et al., 2008). The annotated esterase (corresponding to locus tag BB8809_1755) from *B. longum* subsp. *longum* NCIMB 8809 was selected for analysis and designated *caeA* (for cinnamoyl acid esterase A, its function as will be outlined below). HHPred-based analysis predicts that the CaeA protein shares a conserved structure with esterases from several bacterial species, while BlastP searches indicated that CaeA contains a conserved alpha-beta hydrolase domain which is typical of esterases (Nardini and Dijkstra, 1999).

Sequence alignment of CaeA with several experimentally validated esterases, including an esterase from *Bifidobacterium animalis* subsp. *lactis* with activity against chlorogenic acid (Raimondi et al., 2015), showed the presence of the conserved Gly–X–Ser–X–Gly esterase hydrolytic motif around the Ser-His-Asp catalytic triad. The active site Ser is at the centre of the Gly-X-Ser-X-Gly motif (Supplementry Figure 1). However, these esterases exhibit low sequence similarity to CaeA, ranging from 27 to 33%. CaeA is predicted to represent a cytoplasmic protein as based on SignalP prediction (Petersen et al., 2011). Since the *caeA* gene is located within a genetic locus presumed to be involved in arabinoxylan and arabinan metabolism, we speculate that CaeA may be involved in the removal HCAs from the arabinose residues in arabinoxylan, arabinan and perhaps other plant carbohydrates. For this reason we wanted to confirm the suspected esterase activity of CaeA against model HCA substrates.

### Heterologous Expression and Hydrolytic Activity of CaeA

**FIGURE 2:**
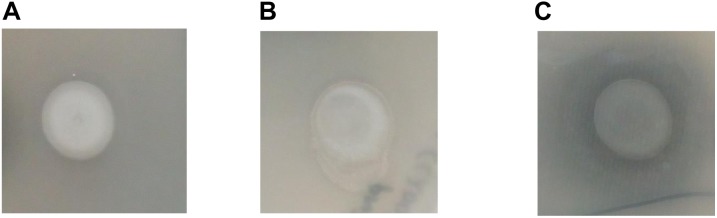
Growth of *B. breve* UCC2003 **(A)**, *B. breve* UCC2003 pNZ44 **(B)** and *B. breve* UCC2003 pNZ44caeA **(C)** on RCA supplemented with 0.1% (v/v) ethyl ferulate acid. A zone of clearing indicates esterase activity.

In order to assess if CaeA is able to hydrolyse ethyl ferulate, a model substrate for esterase activity (Donaghy et al., 1998; Ramos-De-La-Peña, 2016),*caeA* was cloned into the expression vector pNZ44 (McGrath et al., 2001), to generate pNZ44caeA, and introduced into *Bifidobacterium breve* UCC2003 which does not contain a *caeA* homolog. *B. breve* UCC2003 WT, *B. breve* UCC2003 pNZ44 (negative control) and *B. breve* UCC2003 pNZ44caeA were then spot plated on to RCA supplemented with 0.1% (vol/vol) ethyl ferulate and a zone of clearance was observed arround the spotted colonies, indicating the breakdown of ethyl ferulate in the case of *B. breve* UCC2003 pNZ44caeA, indicating expression of esterase activity supplied by the CaeA protein, yet not for *B. breve* UCC2003 WT or *B. breve* UCC2003 pNZ44 (Figure 2). This result therefore supports the notion that CaeA is a functional esterase capable of hydrolysing ethyl ferulate.

### Protein Purification of CaeA and Enzymatic Activity Against Model HCA Substrates

**FIGURE 3:**
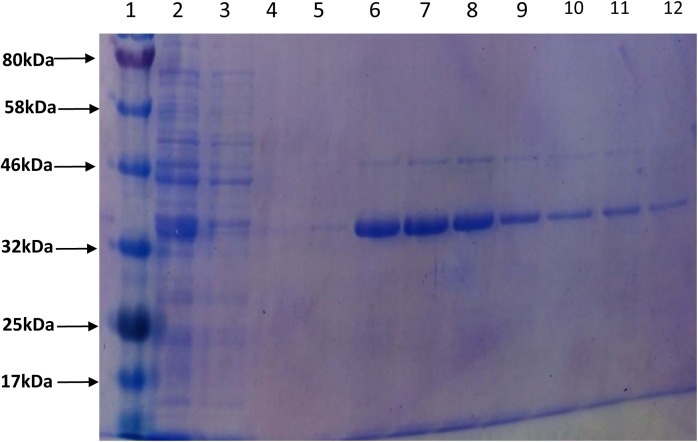
Purification of CaeA. The 12.5% SDS-PAGE gel including protein standard ladder (lane 1), supernatant (lane 2), column wash (lane 3), column wash (lane 4) and elution aliquots (lanes 5 – 12).

**FIGURE 4:**
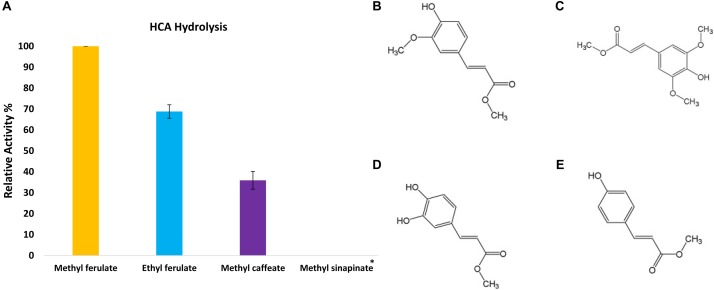
Release of HCAs from the methyl ester substrate. **(A)** The relative activity of CaeA against synthetic HCA esters; methyl ferulate, ethyl ferulate and methyl caffeate. Assays were performed in 1 mM NaH_2_PO_4_/K_2_HPO_4_ pH 7 at 37°C for 2 h with a protein concentration of 6 μg/ml. Data is representative of mean values and standard error of the mean. ^∗^No measurable enzyme activity was found against methyl sinapinate. **(B)** The structure of Methyl ferulate. **(C)** Methyl sinapinate and **(D)** Methyl Caffeate **(E)** Methyl *p*-Coumarate. These structures were partially adapted from a previous publication (Ramos-De-La-Peña, 2016).

**FIGURE 5:**
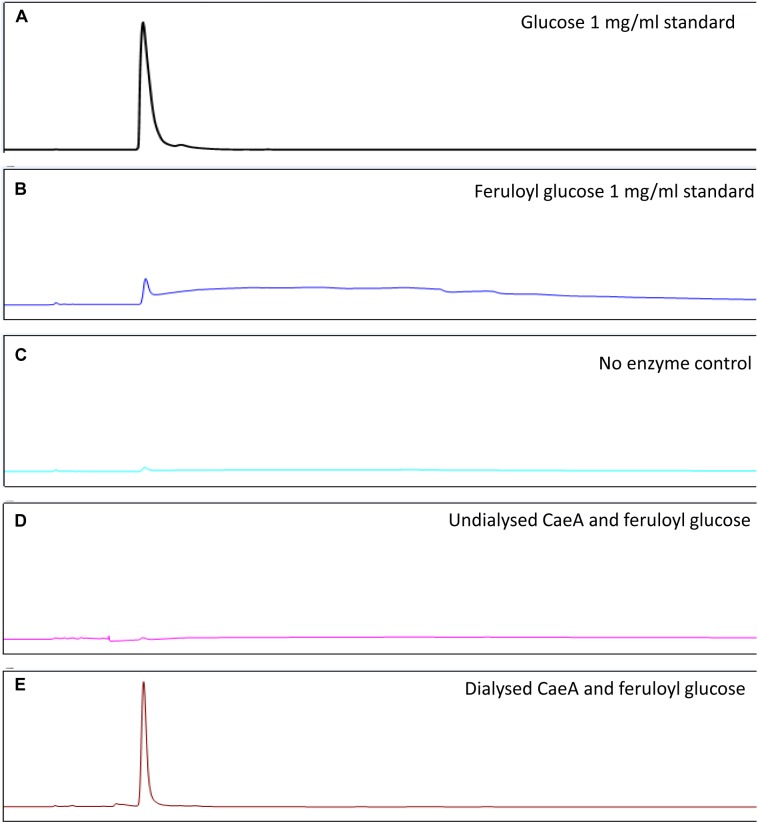
HPAEC – PAD analysis of CaeA activity against feruloyl glucose. **(A)** Glucose standard (1 mg/ml). **(B)** Feruloyl glucose (0.5 mg/ml) standard. **(C)** No enzyme control where feruloyl glucose is incubated for 16 h at 37°C. **(D)** Undialysed CaeA incubated with feruloyl glucose after 16 h at 37°C. **(E)** Dialysed CaeA incubated with feruloyl glucose after 16 h at 37°C. Assays were carried out in 0.1M sodium phosphate buffer at pH 7.5 with 15 μg/ml of protein.

In order to assess the enzymatic activity and substrate specificity of CaeA, a His-tagged version of this protein was expressed in *L. lactis* NZ9000 and purified by Ni–affinity chromatography. This His-tagged CaeA protein was shown to exhibit an approximate size of 36 kDa when analysed by SDS-PAGE (Figure 3), in agreement with the molecular mass (35.57 kDa) of the protein including the N-terminal His_10_-tag as determined by the ExPASY molecular weight calculator (Bjellqvist et al., 1994). An additional band, presumed to be a co-eluted protein, is observed in the gel just above the CaeA protein band. For this reason we used a negative control in all enzyme assays described below, represented by a nisin-induced *L. lactis* NZ9000 culture carrying the empty expression vector. The purified His-tagged CaeA protein was tested for esterase activity against several substrates (i.e., methyl ferulate, ethyl ferulate, methyl caffeate, methyl *p*–coumaric acid, and methyl sinapinate) to determine substrate specificity, and to assign CaeA to either of the esterase sub-groups A, B, C, or D. CaeA was shown to release the associated HCA from methyl ferulate, ethyl ferulate, methyl *p*–coumaric and methyl caffeate, while no noticeable activity was found against methyl sinapinate (Table 3 and (Supplementary Figure S2). These results indicate that CaeA can be classified as a type B feruloyl esterase (Crepin et al., 2004). A subsequent assay was employed to quantify the amount of HCA released once the ester bond of the HCA esters is hydrolysed. The obtained results demonstrate that CaeA can release HCA from methyl ferulate, ethyl ferulate and methyl caffeate, while there was no detectable activity against methyl sinapinate. Methyl *p*-coumaric acid and chlororgenic acid were also tested, however; due to the intrinsic properties of these substrates HCA release could not be accuratley measured in this assay. CaeA was most active toward methyl ferulate under these conditions (Figure 4). This contrasts with the activity of the esterase from *Lactobacillus plantarum* WCFS1, which was shown to exhibit more activity toward methyl caffeate (Esteban-Torres et al., 2013). CaeA was able to cleave methyl caffeate, yet was less efficient with a relative activity of 36% as compared to 68% activity toward ethyl ferulate. CaeA was furthermore shown to cleave the ester bond of 6-*O*-feruloyl glucose, thereby releasing glucose as detected by HPAEC–PAD (Figure 5).

**Table 3:** HPLC analysis of CaeA activity against HCA substrates.

HCA substrate	Activity
Methyl ferulate	**+**
Ethyl ferulate	**+**
Methyl *p*-coumaric acid	**+**
Methyl caffeate	**+**
Methyl sinapinate^∗∗^	**-**


*^∗∗^No hydrolysis evident in the case of Methyl Sinapinate.*

### Esterase Versus Lipase Substrate Range of CaeA

**FIGURE 6:**
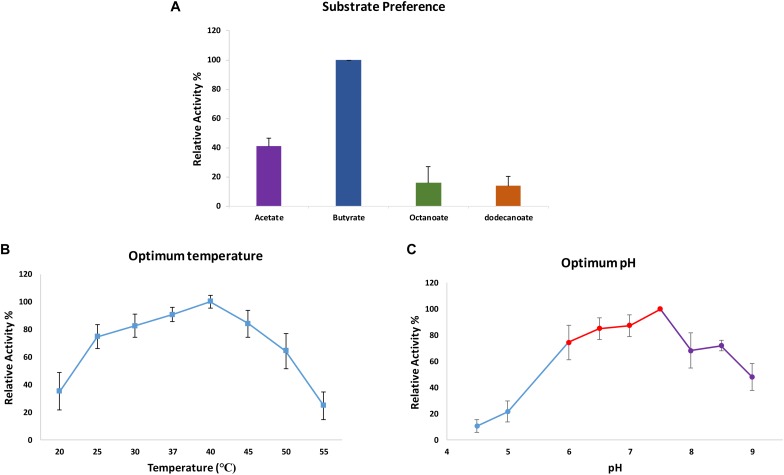
Determination of substrate specificity, pH optimum and temperature optimum of CaeA. Maximal observed activity on *p*-Np butyrate was defined as 100%. **(A)** Optimum substrate preference assays were carried out in 0.1 M NaH_2_PO_4_/K_2_HPO_4_ buffer pH 7.5 at 37°C. **(B)** Optimum temperature assays were performed in 0.1 M NaH_2_PO_4_/K_2_HPO_4_ buffer pH 7.5 at various temperatures to ascertain the optimum temperature for CaeA. **(C)** Optimum pH assays were performed at 37°C in 0.2 M Citric phosphate buffer (Blue), 0.1 M NaH_2_PO_4_/K_2_HPO_4_ buffer (Red) and 50 mM Tris-HCL buffer (Purple). All assays were carried out with *p*-Np butyrate as the substrate. Data is representative of mean values and standard error of the mean.

The hydrolytic activity of CaeA toward several colorimetric substrates containing 4–12 carbons was also determined. ‘True’ esterases generally recognize substrates that contain less than six carbons, whereas lipases may be active on substrates containing more than six carbons (Bornscheuer, 2002). The activity in these colorimetric assays was determined by the amount of released *p*-Np using a photospectrometer at 348nm. The mean specific activity of CaeA on *p*-Np butyrate is 8.35 μmol min^-1^mg^-1^. The activity of CaeA toward *p*-Np acetate, *p*-Np octanoate, and *p*-Np dodecanoate was then determined relative to that observed for *p*-Np butyrate (which was set at 100%) (Figure 6). From the obtained results it is clear that CaeA has a substrate preference for *p*-Np butyrate and appears to be functioning as a ‘true’ esterase since the enzyme elicits substantially reduced activity toward the longer chain substrates with just 13.7% and 15.7% activity against *p*-Np octanoate (8 carbons in length) and *p*-Np-dodecanoate (12 carbons in length), respectively. CaeA also exhibits a lower relative activity of 40.8% toward *p*-Np acetate. In contrast, other esterases from several lactobacilli species and *B*. animalis subsp. *lactis* DSM 10140 have been shown to exert maximal hydrolytic activity toward the shorter *p*-Np acetate, though exhibit low activity toward *p*-Np octanoate, a property they have in common with CaeA (Fritsch et al., 2017). Nonetheless, CaeA is not unique in exhibiting its preferred actitivy toward *p*-Np butyrate (Esteban-Torres et al., 2013, 2015).

### Optimum pH, Temperature and Effect of Ions on CaeA

**FIGURE 7:**
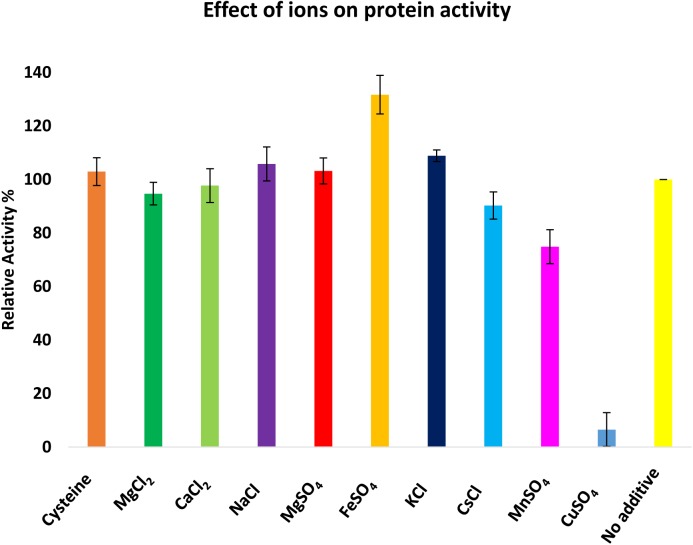
Investigation of the effect of ions on the activity of CaeA. Ions were added at a 1 mM final concentration and 100% activity was defined as the activity of CaeA in the absence of any additive. Assays were performed at 37°C in 0.1 M NaH_2_PO_4_/K_2_HPO_4_ buffer pH 7.5 using *p*-Np butyrate as a substrate. Data is representative of mean values and standard deviation. Maximal activity was defined as 100%.

The biochemical properties of CaeA were investigated to ascertain the reaction conditions for optimal activity of CaeA. The optimum temperature and pH were determined by measuring the release of *p*-Np, a colourimetric substrate at 348 nm, from *p*-Np butyrate. Relative activity for each condition was calculated by normalising the data to the highest specific activity of CaeA, 12.65 μmol min^-1^mg^-1^ for pH and 25.40 μmol min^-1^mg^-1^ for temperature, and expressing the data as a percentage relative to this value. The optimal temperature for CaeA was found to be 40°C and the optimum pH was 7.5 (Figure 6). The lowest activity of CaeA was observed at 55°C and pH 4.5, conditions that diminished activity to 25% and 11%, respectively. Nonetheless, CaeA appears to be a versatile enzyme, exhibiting activity across a rather wide range of temperatures and pH conditions. The effect of ions and detergents on CaeA was also investigated (Figure 7). No substantial impact on esterase activity was noted except for the addition of Cu^2+^ which reduced activity to 7%. Reduction of esterase activity by Cu^2+^ has been reported elsewhere in the literature (De Santi et al., 2016; Esteban-Torres et al., 2016; Xu et al., 2017).

## Discussion

Members of the *B. longum* subsp. *longum* taxon have been specifically associated with complex plant carbohydrate metabolism, making these plant-derived glycans candidate prebiotics for these bifidobacteria. HCAs are frequently found esterified to plant carbohydrates that are indigestible to the human host and are therefore more likely released in the colon by particular members of the gut microbiota (Kroon et al., 2000; Andreasen et al., 2001; Russell et al., 2008; McLaughlin et al., 2015; Ozcan et al., 2017). Much work on plant-derived poly/oligosaccharide metabolism in bifidobacteria has focussed on arabinofuranosidase, xylanase and β-glucosidase activities (Gueimonde et al., 2007; Pokusaeva et al., 2011; Fujita et al., 2014; Zhu et al., 2014; Ku et al., 2015). However, since HCAs are a component of plant carbohydrates it is also important to investigate if bifidobacterial produce esterases.

In the current study we identified and biochemically characterised a novel HCA esterase from *B. longum* subsp. *longum* NCIMB 8809. Significantly, this esterase-encoding gene was located within a highly conserved locus within the genome of all analysed members of this taxon. The *B. longum* subsp. *longum* taxon is known to metabolise plant oligosaccharides such as arabinoxylan and arabinan (Riviere et al., 2014), and therefore the genetic location of this esterase in an arabinoxylan/arabinan metabolism cluster suggests that HCAs that are attached to the arabinose residues of plant carbohydrates are cleaved off as part of the process of metabolising these complex plant cell wall carbohydrates (Schell et al., 2002; Arboleya et al., 2018). This co-location of an esterase-encoding gene within a polysaccharide utilisation locus is similarly reported for other species of bacteria in the gut microbiota such as *Bacteroides* species (Despres et al., 2016; Ndeh et al., 2017). Furthermore, these plant cell wall glycans have been reported to act as prebiotics stimulating bifidobacterial growth in the gut (Vardakou et al., 2007; Neyrinck et al., 2011; Truchado et al., 2015). In order to allow enzymatic access to these dietary polysaccharides bifidobacteria are likely to require an esterase to remove HCAs prior to the metabolism of the carbohydrate moiety. Nonetheless, Riviere and colleges found that the presence or absence of an esterase gene in bifidobacterial strains did not correlate to a strain’s ability to metabolise arabinoxylo-oligosaccharides (Riviere et al., 2014). It must be noted that the actual specific constituents of arabinoxylan and AXOS are highly variable (Arboleya et al., 2018; Riviere et al., 2018), and that an esterase may therefore not be needed by all strains to metabolise certain AXOS constituents.

We demonstrated that the purified CaeA esterase was active against a number of different substrates, such as feruloyl glucose and *p*-Np butyrate. Heterologous expression of CaeA in *B. breve* UCC2003 also conferred esterase activity to a bifidobacterial strain normally devoid of esterase activity. CaeA contains the general characteristic esterase G-X-S-X-G motif, Ser – Asp – His catalytic triad and the conserved alpha/beta hydrolase structure typical of esterase and lipases. CaeA is a ‘true’ esterase rather than a lipase as it elicits a preference for smaller carbon backbone substrates less than six carbons. It has previously been reported that bifidobacterial esterases from *B. animalis* subsp. *lactis* WC 0432 and *B. animalis* subsp. *lactis* DSM 10140 exhibit hydrolytic activity against chlorogenic acid and artificial HCA-containing substrates (Raimondi et al., 2015; Fritsch et al., 2017).

Certain bifidobacterial taxa may be able to release HCAs from plant oligosaccharides in the gut and may make these phenolic compounds available for their own metabolic use, to the human host and/or to other members of the gut microbiota. HCAs have been reported to act as external electron acceptors and may thus provide an energetic advantage for heterofermentative lactobacilli by increasing the amount of ATP and NADH regeneration (Filannino et al., 2014; Filannino et al., 2016). Increased bioavailability of the HCAs may also have consequences and/or reflect the disease state of the host. In diabetes-resistant rat models lactobacilli and bifidobacteria were found to be more abundant compared to diabetes-sensitive rats (Roesch et al., 2009); lactobacilli with an increased capability of HCA hydrolysis were isolated from the same patient sample set (Lai et al., 2009). However, it should be noted that conclusive proof for HCA metabolism by bifidobacteria is as yet lacking.

Similar to the esterase from *B. animalis* subsp. *lactis* WC0432, CaeA is presumed to be an intracellular enzyme as based on the lack of an obvious protein secretion signal (Raimondi et al., 2015). Therefore, whether certain bifidobacteria increase bioavailablity of HCAs to the host still remains unclear. A limitation of our study is that we did not employ plant oligosaccharide substrates substituted by HCAs to test this as the plant oligosaccharide isolation process usually removes HCAs. Future work should determine if bifidobacteria can metabolise HCAs, and if so, assess the consequences of this ability for bifidobacterial physiology in the gut environment. Furthermore, the question should be addressed as to whether or not bifidobacteria release HCAs in their environment to make them available to the host or other gut microbes.

## Conclusion

This study has found that members of the *B. longum* subsp. *longum* taxon possess a highly conserved esterase-encoding gene, which is co-located with genes associated with plant poly/oligosaccharide degrading enzymes on the *B. longum* subsp. *longum* genome. Therefore, CaeA is likely an important enzyme in the metabolism of plant oligosaccharides by *B. longum* subsp. *longum* taxon. CaeA is a true esterase capable of cleaving several HCA and esterase model substrates and thus bifidobacteria a likely can release HCAs from plant oligosaccharides. *B. longum* subsp. *longum* is the second known bifidobacterial species able to express an esterase that may remove HCAs from plant carbohydrates.

## Author Contributions

SK designed the experiments, carried out the experiments, analysed the experimental data, and wrote the manuscript. JO designed the experiments, carried out the experiments, and analysed the experimental data. MK designed the experiments, carried out the experiments, and analysed the experimental data. DS designed the experiments and wrote the manuscript.

## Conflict of Interest Statement

The authors declare that the research was conducted in the absence of any commercial or financial relationships that could be construed as a potential conflict of interest.

## Supplementary Material

Figure S1 Multiple sequence alignment of CaeA (B8809_1755) from *B. longum* subsp. *longum* NCIMB 8809, Balat_0669 from *B. lactis* subsp. *animalis*, Lp_1002 from *Lactobacillus plantarum* WCFS1, Lp_2923 from *L. plantarum* WCFS1 and lj0536 from *Lactobacillus johnsonii* N6.2. The (Gly – X – Ser – X – Gly) esterase motif is highlighted in the red box and the Ser – Asp – His triad residues are indicated by the red triangles.

Figure S2 HPLC analysis of the activity of CaeA against hydroxycinnaminic acid substrates. Enzyme reactions were carried out in 20 mM morpholinepropanesulfonic acid (MOPS) pH 7.5 with the substrates present at a 1 mM final concentration and 15 μg of CaeA protein per reaction in a final reaction volume of 1 ml. All substrates were also incubated in buffer without CaeA as a negative control. All reactions and negative controls were incubated at 37°C for 16 h and were terminated by the addition of 370 μl ethyl acetate. Assays were performed in duplicate. **(A)** CaeA activity against methyl ferulate and corresponding no enzyme control, **(B)** ethyl ferulate and no enzyme control, **(C)** methyl *p*-coumaric acid and no enzyme control, **(D)** methyl sinapinate and no enzyme control, and **(E)** methyl caffeic acid and no enzyme control.
