# Supplementary figures and images for: Characterisation of a Hydroxycinnamic Acid Esterase From the Bifidobacterium longum subsp. longum Taxon
Source: Front Microbiol. 2018 Nov 9;9:2690. doi: 10.3389/fmicb.2018.02690 (PMC6237967; doi:10.3389/fmicb.2018.02690)

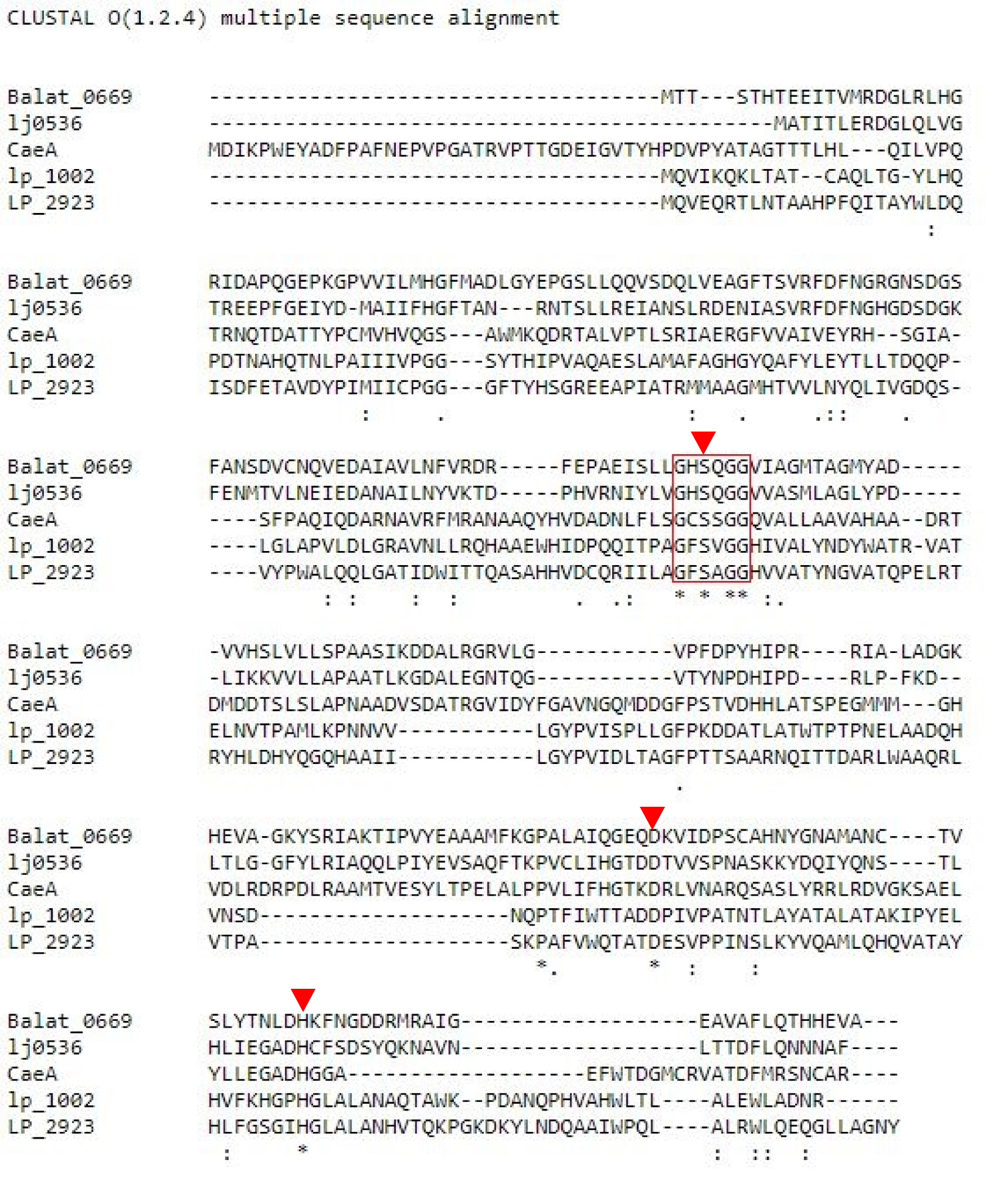

Supplement: Figure S1 — Multiple sequence alignment of CaeA (B8809_1755) from B. longum subsp. longum NCIMB 8809, Balat_0669 from B. lactis subsp. animalis, Lp_1002 from Lactobacillus plantarum WCFS1, Lp_2923 from L. plantarum WCFS1 and lj0536 from Lactobacillus johnsonii N6.2. The (Gly – X – Ser – X – Gly) esterase motif is highlighted in the red box and the Ser – Asp – His triad residues are indicated by the red triangles. [file Image_1.TIF]

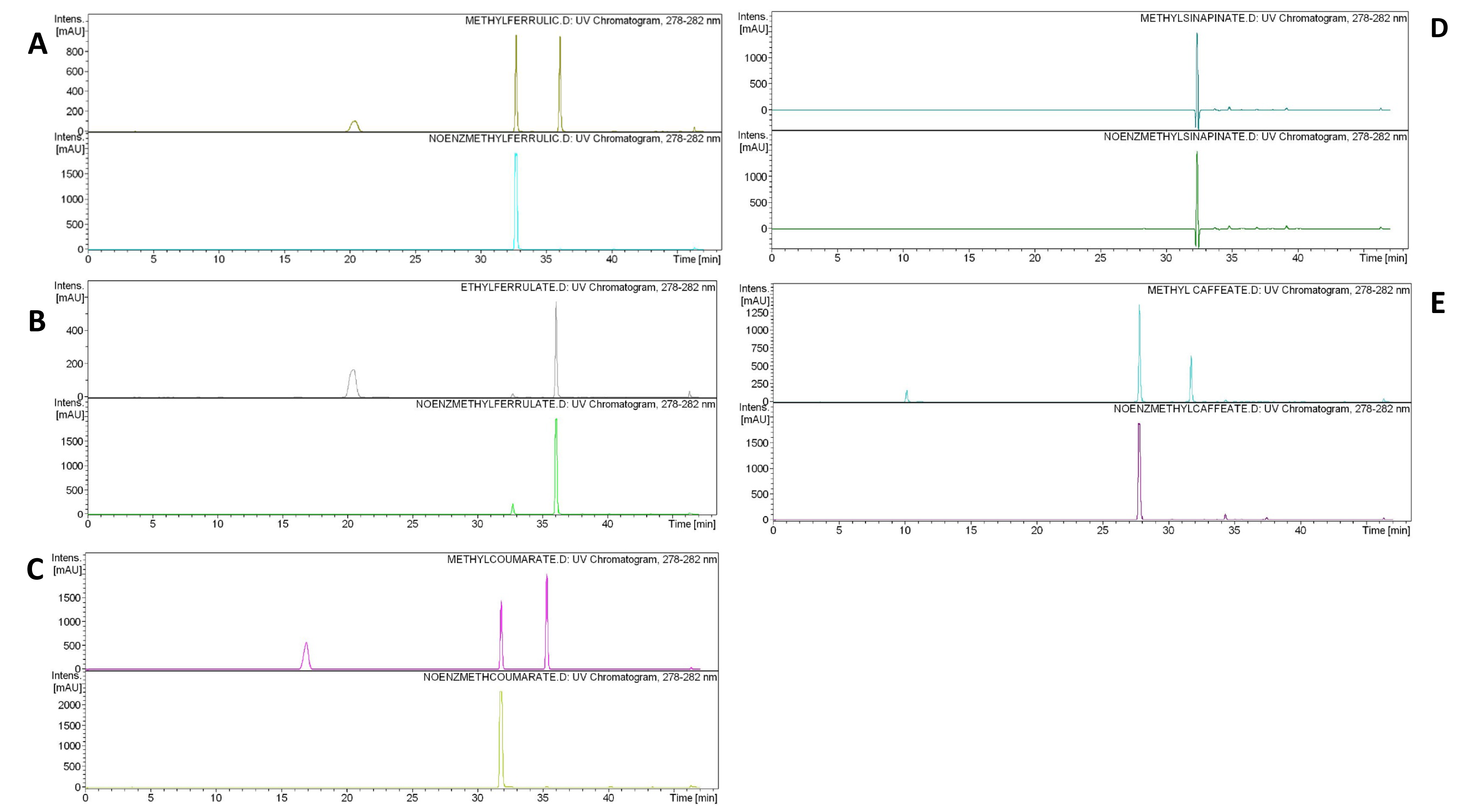

Supplement: Figure S2 — HPLC analysis of the activity of CaeA against hydroxycinnaminic acid substrates. Enzyme reactions were carried out in 20 mM morpholinepropanesulfonic acid (MOPS) pH 7.5 with the substrates present at a 1 mM final concentration and 15 μg of CaeA protein per reaction in a final reaction volume of 1 ml. All substrates were also incubated in buffer without CaeA as a negative control. All reactions and negative controls were incubated at 37°C for 16 h and were terminated by the addition of 370 μl ethyl acetate. Assays were performed in duplicate. (A) CaeA activity against methyl ferulate and corresponding no enzyme control, (B) ethyl ferulate and no enzyme control, (C) methyl p-coumaric acid and no enzyme control, (D) methyl sinapinate and no enzyme control, and (E) methyl caffeic acid and no enzyme control. [file Image_2.TIF]
